# Knowledge, attitudes, and practices of health care waste management among Zambian health care workers

**DOI:** 10.1371/journal.pgph.0000655

**Published:** 2022-06-22

**Authors:** Colleen M. Leonard, Chipwaila Choolwe Chunga, Justine M. Nkaama, Kutha Banda, Chilekwa Mibenge, Victor Chalwe, Godfrey Biemba, Sandra Chilengi-Sakala, Florence Kabinga Mwale

**Affiliations:** 1 National Health Research Authority, Lusaka, Zambia; 2 Ministry of Health, Lusaka, Zambia; Instituto Nacional de Geriatria, MEXICO

## Abstract

Poor management of health care waste poses a serious threat to the health of health care workers, patients and communities. In developing countries, adequate health care waste management (HCWM) is often a challenge. To address this, the Zambian Health Services Improvement Project with HCWM as a component, was implemented in five Zambian provinces (Luapula, Muchinga, Northern, North-Western and Western Provinces), under which this cross-sectional study was conducted to identify the knowledge, attitudes, and practices of health care workers on HCWM. Fifty government hospitals and health posts from five provinces in Zambia were included in the study. Data was collected using a mixed-methods approach, which included surveys with health care workers (n = 394), in-depth interviews (n = 47) with health officials at the provincial, district, and facility levels, and observational checklists (n = 86). Overall, knowledge of proper waste segregation was average (mean knowledge score 4.7/ 7). HCWM knowledge varied significantly by job position (p = 0.02) and not by facility level, years of service, nor prior training. Only 37.3% of respondents recalled having received any sort of HCWM training. Poor waste segregation practice was found as only 56.9% of the facilities used an infectious waste bag (yellow, red or orange bin liner) and a black bag for general waste. This study revealed that only 43% of facilities had a functional incinerator on site for infectious waste treatment. Needle sticks were alarmingly high with 31.3% of all respondents reporting a prior needle stick. The system of HCWM remains below national and international standards in health facilities in Zambia. It is imperative that all health care workers undergo comprehensive HCWM training and sufficient health care waste commodities are supplied to all health facility levels in Zambia.

## Introduction

There has been a rapid growth of health care waste in developing countries over recent years due to increased access to medical services; therefore, proper health care waste management (HCWM) is essential [1]. Poor management of health care waste can lead to adverse health and environmental effects [2,3]. At each stage of the HCWM system (segregation, storage, transport, treatment and disposal), there is potential to spread infectious diseases [2,4]. Poor HCWM can impact the health of health care workers, patients, and even communities, especially in low- and middle-income countries [5]. Furthermore, inadequate treatment of medical waste, such as openly burning waste, poses serious environmental risks through harmful emissions to the surrounding community [6–8].

Health care waste may be categorized as either hazardous or non-hazardous. Hazardous waste consists of infectious materials, sharps, chemical waste, pharmaceuticals, and radioactive waste [4]. Infectious waste includes waste contaminated with blood or other bodily fluids, cultures from laboratory work, and waste items from patients, including but not limited to: bandages, swabs, discarded tissue samples, blood microscopy slides, and disposable medical devices [9]. Non-hazardous waste, such as plastic packaging, paper and office products, is waste that does not pose any biological, chemical, radioactive or physical harm [9]. It is estimated that globally about 15% of the total waste generated in Health Care Facilities (HCFs) is hazardous [9]. Hazardous waste poses occupational health and safety risks, and environmental pollution to the surrounding community if not disposed of properly [2]. Infectious waste contaminated with Human Immunodeficiency Virus (HIV), Hepatitis B and C viruses can pose harm to health care providers. According to the World Health Organization, of the approximate 35 million health workers worldwide, around 3 million (9.4%) are exposed to blood borne pathogens through a percutaneous injury annually (e.g. contaminated needle stick injuries) [10]. Percutaneous injuries among health workers can occur as a result of mishandling sharps as well as poor practices like recapping used needles [10]. Further, in 2000, the WHO indicated that inadequate disposal, handling and reuse/recycling of contaminated syringes and other waste items resulted in 21 million Hepatitis B infections (32% of all new infections), two million Hepatitis C infections (40% of all new infections) and 260,000 HIV infections globally (5% of all new infections) [11]. Poor handling and disposal of medical waste not only impacts the health of health care workers, but also that of patients, visitors, and non-hospital staff involved in the handling and treatment of infectious health care waste. In addition, many developing countries face HCWM burdens; consequently, approximately 50% or more of the global population is exposed to environmental, occupational and public health risks from poor HCWM [5].

In sub-Saharan Africa, the state of HCWM is often below international standards [12,13]. A study in Cameroon, found that health care waste collection and handling systems, including containers and bins for segregated wastes, are generally in a poor state [3]. There is a lack of research on the state of HCWM in Zambia. In Zambia, there are three different health facility levels: health centres/posts, district and regional level hospitals. Each level (and unique facility) has different resources and staff available. Therefore, it is likely that HCWM attitudes and practices vary widely between facilities.

The National Health Care Waste Management Plan seeks to establish a sustainable HCWM system that takes into account environmentally sound practices, principles and commitments, including organizing HCWM options that are technically, socially, and economically appropriate [14]. This aims to reduce the transmission of communicable diseases through proper disposal of health care waste at health care facilities and disposal centres. Improved waste management practices also have important benefits at the national level, which include improved environmental health due to reduced water and soil pollution of nearby communities; creation of job and livelihood opportunities in the area of waste management, treatment and disposal; and a reduction in the overall costs for waste management.

It is important to understand the gaps in attitudes, knowledge and practice surrounding HCWM in Zambia. This study was conducted on a wide scale investigating health care waste management at three different health facility levels within five provinces of Zambia. We conducted a cross-sectional study to identify the knowledge, attitudes, and practices (KAPs) of health care workers on HCWM and to explore the individual and institutional factors associated with proper HCWM practices. To our knowledge, this is the first published study of its kind assessing the knowledge, attitudes and practices of HCWM in health facilities across various provinces in Zambia.

## Materials and methods

This cross-sectional, mixed-methods study was conducted in November 2018 in five Zambian provinces (Luapula, Muchinga, Northern, North-Western and Western Provinces). These provinces were chosen because the Zambian Health Services Improvement Project (ZHSIP) was being implemented there. A two-stage sampling method was used to select the study hospital and health centers in each province. The sampling frame included all public hospitals at each level, including rural and urban health centres and health posts. In the first stage, two districts in each province were selected using simple random sampling (SRS). In the second stage, the main hospital in each district was purposively selected, then four hospitals or health facilities were selected per district using SRS. This amounted to five facilities per district (10 per province) for a total of 50 facilities visited. One health facility in Northern Province and two facilities in Muchinga Province were purposively selected due to logistical challenges attending other facilities.

### Study design and participants

This mixed-methods study had three parts: 1) survey with health care workers to determine their knowledge, attitudes and practices surrounding HCWM (S1 Text); 2) in-depth interviews with health officials at the health facility, district and provincial levels to uncover the attitudes towards HCWM and supplement the quantitative data collected from the surveys (S2 Text); 3) HCWM facility checklist to observe the current practice of HCWM (S1 Checklist). For the survey, health care workers, including doctors, nurses, lab technicians, community health workers, clinical officers, and environmental health specialists were targeted. For the hospitals, multiple wards were visited and a sample of at least five health care workers from each visited ward were surveyed. At a minimum, the lab was visited at each hospital. However, for the urban and rural health centers, all health care workers present were surveyed, including the facility in-charge, nurse(s), environmental health technologist, and community health volunteers (if applicable). The targeted sample size was 410 surveys, which includes a 6% non-response rate (S3 Text). For the in-depth interviews, key respondents that work directly or oversee waste management in the province, district and facility levels were purposively selected. At the provinces and districts, the Provincial Health Director, District Health Director, Environmental Health Officer and Health Promotion Officers were interviewed. At the hospitals and health centres, the facility in-charge and Environmental Health Specialists were interviewed. A total of 50 interviews were targeted. Lastly, the facility checklist was used in all the 50 targeted health facilities. For all health centers and health posts, the checklist was administered once whereas for larger health facilities it was administered in each ward. In total, 86 checklists were completed.

### Data collection and management

Data collection was conducted by five survey teams, one for each province. The survey data was collected using Open Data Kit (ODK) on a portable tablet, which allows for real-time electronic data capture. Each survey was conducted in-person, on site, with one enumerator and one respondent at a time. The survey can be viewed in the Supplementary materials (S2 Text). For the in-depth interviews, a paper in-depth interview guide was used (S3 Text). All interviews were recorded and transcribed, unless the interviewee declined to be recorded. Lastly, a checklist was used to observe the HCWM practices and HCWM items present at each facility (S4 Text). The checklist was a spot checklist to record if various waste management items were available and/or functional. At each hospital, one checklist was completed for each ward visited. Participant confidentiality was ensured and no names or identifiable markers were recorded on the data collection forms. Each survey team had a data manager. The data manager was responsible for making sure all data from each tablet was uploaded into the secure server at the end of each day. The data manager also conducted random spot checks with the survey data to flag any mistakes or inconsistencies in the data. The original dataset was password protected and stored on a secure computer.

### Data analysis

Descriptive statistics were conducted, including chi-square tests. Chi-square tests of independence were used to compare outcomes across various factors including facility type, position, and years of service. If assumptions were not met, Fischer’s exact test were utilized. A composite variable called knowledge on waste management was derived based on the wastes that belong either in the yellow bag, sharp container, or black bag. Respondents that were able to allocate at least six out of the seven hospital waste locations correctly were classified as having high knowledge on waste management, those who could not were classified as having low knowledge. All statistical analyses were performed using Stata 13 (StataCorp, College Station, Texas, USA) and Microsoft Excel (2016).

For the qualitative analysis of the in-depth interviews, all interview recordings were transcribed by research assistants. Thematic analysis was conducted to identify key factors that were associated with waste management in the health facilities. Four codes were formed (level of knowledge, attitude and practice; socio-demographic factors associated with HCWM; institutional factors; and adherence to policies) to index and identify key emerging themes of the interviews.

### Ethics statement

Ethical clearance was obtained from the University of Zambia Biomedical Research Ethics Committee (UNZABREC) and the National Health Research Authority (NHRA) of Zambia. Written informed consent was obtained from each participant prior to starting the survey or interview. All participants were given a copy of the consent form to keep for their own records.

## Results

In total, 50 facilities were visited (26% in urban areas). A total of 394 respondents participated in the KAPs survey (Table 1). Most of the respondents were female (58.6%) and the slightly over half of the respondents (51.3%) were from the district hospitals. Nursing was the most common field represented at 36.3% of those surveyed, and the next most common group was the cleaning staff at 13.2%. The lowest representation was from doctors which comprised 2% of all respondents. Half of the respondents were between 20–29 years old. Lastly, the majority of all respondents (59.1%) had one to four years of experience in their current position.

**Table 1:** Characteristics of the survey respondents.

	Survey respondents
	N = 394 n (%)
**Province**	
Luapula	77 (19.5)
Muchinga	70 (17.7)
North Western	84 (21.3)
Northern	85 (21.6)
Western	78 (19.8)
**Type of Facility**	
District Hospital	201 (51.3)
Regional Referral Hospital	31 (7.9)
Health centre/Health post	161 (40.9)
**Position**	
Community Health Assistant	37 (9.4)
Clinical Officer	28 (7.1)
Environmental Health Staff	24 (6.1)
Midwife	14 (3.6)
Cleaner	52 (13.2)
Doctor	8 (2.0)
Laboratory Staff	37 (9.4)
Nurse	143 (36.3)
Other	50 (12.7)
**Gender**	
Male	163 (41.4)
Female	231 (58.6)
**Years of Service**	
<1	25 (6.4)
1–4	233 (59.1)
5–9	63 (16.0)
10+	73 (18.5)
**Total respondents**	394 (100)

### Knowledge

A survey question was administered asking where seven different waste items should be disposed (S2 Text). If the respondent answered six or seven correct, then they were deemed to have “high knowledge” of the proper health care waste management practices. Any score lower than six was deemed “low knowledge.” Luapula Province had the highest proportion of respondents (47%, n = 36) with a high knowledge of proper HCWM practices. On the other hand, North-western and Western Provinces had the lowest proportion of respondents with a high knowledge of proper HCWM practices at 26%. Overall, the knowledge of proper HCWM practices was average. The mean knowledge score was 4.7 out of 7 (SE = 0.07). In terms of individuals having a high knowledge of HCWM practices, only 34% of all respondents had a high level of knowledge. The knowledge of waste segregation by waste item was average for all items except the empty intravenous (IV) bag, for which only 24% of respondents knew that the item should be discarded in the domestic bin (black bin liner) (Table 2). Overall, waste segregation knowledge was found to be associated with the position of the health worker (Table 3). For the health workers, there was a wide range of knowledge with the laboratory staff having the highest knowledge and midwives having the lowest. By health facility type, health workers from the regional hospitals were the most knowledgeable and those from the health centres/ health posts had the lowest level of waste disposal knowledge; however, this trend was not statistically significant. Prior training in HCWM was not found to be associated with knowledge of HCWM.

**Table 2:** Knowledge of correct health care waste disposal practice by individual item.

Waste Item	Correct answer	Answered correct n(%)
Blood saturated gauge	Yellow/red bag	322 (81.7%)
Empty IV bag	Black bag	95 (24.1%)
Used hypodermic needle	Sharps container	341 (86.6%)
Suction canister with body fluids	Yellow/red bag	283 (71.8%)
Broken mercury thermometer	Sharps container	277 (70.3%)
Used gloves	Yellow/red bag	273 (69.3%)
Leftover food	Black bag	276 (70.1%)
**All Correct**		28 (7.1%)
**High knowledge (≥ 6 correct)**		134 (34.0%)

**Table 3:** Waste segregation knowledge by facility type, job position, years of service, and prior training in health care waste management.

	High Knowledge	p-value
**Type of Facility**		0.122
Regional Referral Hospital (n = 110)	45 (40.9%)	
District Hospital (n = 109)	38 (34.9%)	
Health centre/Health post (n = 175)	51 (29.1%)	
**Position**		0.021*
Community Health Assistant (n = 37)	12 (32.4%)	
Cleaning staff (n = 53)	12 (22.6%)	
Clinical Officer (n = 28)	8 (28.6%)	
Doctor (n = 8)	1 (12.5%)	
Environmental Health Staff (n = 24)	7 (29.2%)	
Laboratory Staff (n = 37)	16 (43.2%)	
Midwife (n-14)	0 (0%)	
Nurse (n = 143)	60 (42.0%)	
Other (n = 50)	18 (36.0%)	
**Years of service** ^**a**^		0.273
<1 (n = 25)	5 (20.0%)	
1–4 (n = 233)	86 (36.9%)	
5–9 (n = 63)	18 (28.6%)	
10+ (n = 73)	25 (34.3%)	
**Training in HCWM**		0.380
Yes (n = 147)	46 (31.3%)	
No (n = 247)	88 (35.6%)	

^a^In their current position.

*Statistically significant at alpha = 0.05.

In terms of training, only 37.3% of respondents recalled having received any sort of waste management training. Like knowledge of HCWM practices, prior training was found to be associated with the position of the health worker. For example, most environmental health staff and cleaners (71% and 50%, respectively) stated that they had received waste management training in the past. For all other positions, less than half of the workers had received waste management training (Table 4).

**Table 4:** Training in health care waste management by job position and years of service.

	Received n(%)	Not received n(%)	p-value
**Type of Facility**			0.140
Regional Referral Hospital	47 (42.7%)	63 (57.3%)	
District Hospital	44 (40.4%)	65 (59.6%)	
Health centre/Health post	56 (32.0%)	119 (68.0%)	
** Position**			0.001*
Nurse	51 (35.7%)	91 (64.3%)	
Doctor	0 (0%)	8 (100%)	
Environmental Health Staff	17 (70.8%)	7 (29.2%)	
Cleaner	26 (50%)	26 (50%)	
Community Health Assistant	16 (43.2%)	21 (56.8%)	
Clinical Officer	5 (18.5%)	22 (81.5%)	
Laboratory Staff	12 (32.4%)	25 (67.6%)	
Midwife	6 (42.9%)	8 (57.1%)	
Other	14 (27.5%)	37 (72.5%)	
**Years of Service**			0.462
Less than 1 year	6 (24%)	19 (76%)	
1–4 years	89 (38.2%)	144 (61.8%)	
5–9 years	22 (34.9%)	41 (65.1%)	
10+ years	30 (41.1%)	43 (58.9%)	

*Statistically significant at alpha = 0.05.

### HCWM practice

From the checklist, we assessed the presence of various HCWM items (Table 5). Eighty-six observational checklists were completed in total. When visiting the health facilities and hospital wards, we found that most had bins for segregating waste (89.5%). Yet, bin liners were less common. Only 61.6% of facilities had yellow or red biohazard bin liners for hazardous waste. Even fewer had black bin liners for domestic waste (41.9%). However, in practice, 94% of the staff interviewed say that they segregate different types of waste (Table 5). Almost all facilities and wards (94.2%) had a sharps box on site for the safe disposal of needles and syringes. For safe treatment and disposal of infectious waste, only 43.2% of facilities had a functional incinerator in a secured area on site. For the waste bins, using containers with a lid and bin liner was generally low. It was found to be associated with the type of facility, with the lowest compliance in the district hospitals at 47.7%. Exclusively disposing of infectious waste in a container with a yellow, red, or orange bin liner was also associated with the type of facility, but the highest rate was among the district hospitals (Table 6). In addition, full personal protective equipment (PPE) for waste management staff, timely emptying of storage facilities, and waste transport methods were found to be associated with the type of facility. The regional hospitals were significantly more likely to have full PPE for their waste management staff than the other facility levels. The majority of health facilities stated that their waste storage facility is emptied within 24 hours, but this percentage was the lowest for the health centres/ health posts. For transporting waste to the disposal site, using a wheelchair was the most common alternative method used.

**Table 5:** Presence of various waste management items at the health facility or ward level.

Items	Regional Referral Hospital (n = 19)	District Hospital (n = 14)	Health centre/ Health post (n = 53)	All facilities and wards (n = 86)
Bins for disposing of infectious waste^a^	17 (89.5%)	14 (100%)	46 (86.8%)	77 (89.5%)
Bins for disposing of non-infectious waste^a^	19 (100%)	13 (92.9%)	45 (84.9%)	77 (89.5%)
Yellow/ red bin liners^a^	13 (68.4%)	12 (85.7%)	28 (52.8%)	53 (61.6%)
Black bin liners	12 (63.2%)	8 (57.1%)	16 (30.2%)	36 (41.9%)
Sharps box on site^a^	19 (100%)	13 (92.9%)	49 (92.5%)	81 (94.2%)
Secured waste storage area	15 (79.0%)	10 (71.4%)	28 (53.9%)	53 (61.6%)
	**Regional Referral Hospital (n = 4)**	**District Hospital (n = 5)**	**Health centre/ Health post (n = 35)**	**All facilities**^a^ **(n = 44)**
Functional Incinerator in a secured area on site	4 (100%)	3 (60%)	12 (34.3%)	19 (43.2%)

^a^Health facilities only, not including ward levels.

*Some health facilities were missing completed checklists.

**Table 6:** Waste management practices reported from staff surveys.

	Regional Referral Hospital staff (n = 110)	District Hospital staff (n = 109)	Health centre/ Health post staff (n = 175)	p-value
Facility segregates waste	104 (94.6%)	102 (93.6%)	166 (94.9%)	0.899
Waste management Color scheme				0.126
Yellow/red, black	68 (61.8%)	69 (65.1%)	87 (52.4%)	
Yellow/red, black, brown	7 (0.9%)	1 (6.4%)	9 (5.4%)	
Other	29 (26.4%)	30 (28.3%)	63 (38.0%)	
None	6 (5.5%)	6 (5.7%)	7 (4.2%)	
Dispose of waste in closed container with bin liner	81 (73.6%)	52 (47.7%)	121 (69.1%)	< 0.001*
Exclusively place infectious waste in a container with a yellow, red, or orange bin liner	56 (50.9%)	73 (67.0%)	116 (66.3%)	0.016*
Full PPE^a^ for waste management staff	62 (60.8%)	41 (40.2%)	54 (34.2%)	0.000*
Storage facility is emptied within 24 hours	100 (90.9%)	103 (94.5%)	142 (81.1%)	0.012*
**Waste transport method to disposal site** ^**b**^				
Wheeled bin	35 (31.8%)	14 (12.8%)	13 (7.9%)	< 0.001*
Bins transported by hand by health workers	40 (36.4%)	70 (64.2%)	150 (90.9%)	< 0.001*
Wheel barrow or trolley	29 (26.4%)	20 (18.4%)	4 (2.4%)	< 0.001*
Other	12 (10.9%)	23 (21.1%)	1 (0.6%)	< 0.001*

a) Full PPE includes coverall, heavy duty gloves, and gum boots.

b) Multiple responses allowed.

*Statistically significant p-value at alpha = 0.05.

Only 4.3% of facilities used the WHO recommended color coding for all health care waste, including a brown bin liner for chemical and pharmaceutical waste. Excluding the brown bin liner, 56.9% of facilities use an infectious waste bag (yellow, red or orange) and a black bag for general waste. However, many people interviewed stated that they often label waste containers if they do not have the correct color-coded bin liners. Health workers cited stock-outs as a reason for not having the correct bin liners all the time.

### Attitude and practice

In total, 47 in-depth interviews were conducted with health officials at the provincial, district, and facility levels. Overall, all persons interviewed had a positive attitude towards HCWM. Those who were not as knowledgeable on HCWM usually expressed a willingness to learn. There were differences in HCWM practice between the three different facility levels (province, district, and health post) and facilities with and without an environmental health technician (EHT). Those interviewed at the provincial and district levels stated that they conduct HCWM trainings in the form of an orientation for new staff. One of the district health officers stated:

*“We ensure that all the staff are given an in-house orientation as they report to a facility to ensure they know how that facility handles waste. We have deliberate policies within our district…I assume even in other districts they have…to ensure the EHT take a leading role in orientation and monitoring the staff handling of waste.”*

Therefore, health officers have policies on HCWM and expect the EHT to take the lead in orienting and monitoring staff at all facility levels to ensure proper waste management. However, when respondents were asked if they had received HCWM training in the past only 37.3% of respondents recalled having received any sort of waste management training. Also, usually only the district and provincial hospitals have EHTs, while the health centres/ posts do not have an EHT due to staffing limitations.

Qualitative interviews suggested that the presence of an EHT in health facilities had a positive influence on HCWM. An in-charge at one health facility indicated that they have an EHT who is very involved, especially in waste management. On the other hand, health workers from facilities that do not have EHTs seemed to be less confident in their HCWM practices One in-charge at a rural health center without an EHT said:

*“So we have put a staff to take care of waste management… umm… we are lacking basic training because what we use is just… uh…common knowledge… . in disposing of. So we are using what we have, but I think we are lacking basic, uhh…training.”*

At the facility level, when there is no EHT, someone is usually assigned to be in charge of waste management. One concern that was identified was that some staff at the rural health centres feel that they are lacking adequate training in health care waste management.

### Consequences of poor HCWM

A history of a needle stick was high with 31.3% of all survey respondents reporting a prior needle stick. Of those who had received a needle stick, 59.5% said that it occurred within the first 24 months on the job. The majority (75.4%) of those who experienced a needle stick stated that they reported the needle stick incident to management for mitigation.

## Discussion

Our study reveals that the system of waste management remains below national and international standards in all health facility levels in Zambia. The in-depth interviews with health officials at the provincial, district, and facility levels provide an understanding of the attitudes and policies in place surrounding HCWM. While the surveys give insight into the individual knowledge, comfort level, and practices of HCWM among health workers working at different health facilities. The observational checklists assessed the availability of essential supplies to implement proper HCWM. Overall, we found that although attitudes towards HCWM were generally positive and policies were available at the provincial levels, HCWM knowledge was average and essential supplies were often lacking.

Almost all facilities had bins for disposing infectious waste; however, waste bins with lids and bin liners were not always present. Bin liners and lids limit the exposure of infectious waste items from getting in contact with health workers and patients [4]. Poor waste segregation practice was found as only 62% of health workers stated that they exclusively place infectious waste in a hazardous waste colored bin. The vast majority of facilities were not compliant with the WHO color scheme for segregating waste, including a brown bin liner for pharmaceutical waste [4]. Similar findings have been reported from other HCWM studies in sub-Saharan Africa [15,16]. For example, in one Nigerian hospital, only 54% of the health workers were aware of or had seen color coded bins [15]. In this study, slightly over half (56.9%) of the facilities use an infectious waste bag (yellow, red or orange bin liner) and a black bag for general waste. The district hospitals and health centres/ posts were less likely to have bin liners and color-coded bins compared to the regional hospitals. Procurement of bin liners and lids for each medical waste bin should be a priority, especially for district hospitals and health centres. The waste transport method also varied by facility level. The preferred method of using a wheeled bin was used in less than half of the facilities and hospital wards. In addition, only 43% of all health facilities had a functional incinerator on site; however, this varied greatly by facility level as all four regional hospitals (100%) had a functional incinerator while only 34% of the health centres had one. The remaining facilities often relied on burning their waste in a brick-and-mortar enclosed area. This can cause damage to the environment and create human health problems. Wastes containing polyvinyl chloride and other plastics in IV and blood bags, tubes, and some syringes when burned produce highly toxic chemicals (dioxins and furans) which can cause cancer, infertility and other serious health problems, such as asthma [1,2,17,18]. In Zambia, burning biomass has occurred for many years and is a major source of air pollution [19]. Further environmental damage from improper burning of medical waste should be avoided, and medical waste incinerators that meet WHO standards should made available to the health facilities.

A proper waste management system consists of appropriate segregation, storage, transfer, and disposal of medical waste [2]. A WHO/UNICEF evaluation found that only 60% of sampled health facilities in the WHO Africa region (with 12 countries represented) had adequate waste management systems in place for the safe disposal of health care waste [12]. Our study found a lower percentage as only 43% of the health facilities had a functional incinerator on site. A study of urban health clinics in Ethiopia found that 61% of the surveyed clinics had poor HCWM practices [13]. We found very similar results with regards to HCWM practice variables collected.

Knowledge of HCWM was average (mean score = 4.7/ 7), but it did vary significantly based on the position of the health worker, with the highest knowledge among the laboratory staff. Other staff members, especially the cleaning staff that directly deal with waste disposal, should be targeted for more comprehensive HCWM training. It was interesting to note that prior training in HCWM was not found to be associated with having a high knowledge of HCWM. This suggests that the trainings the health workers have previously received are either not frequent enough or not adequate for lasting retention. Therefore, more comprehensive training should be given to all health workers at orientation and at regular intervals throughout their post.

In terms of training, previous HCWM training was low and ranged from 32–43%, depending on the health facility level. A similar rate of health workers having received HCWM management training (37%) was found in a study in Ethiopia [20]. In Zambia, an EHT is normally in charge of training health workers in HCWM. The EHT is also responsible for monitoring and implementing a system to correct any errors in disposing health care waste. However, usually only the district and provincial hospitals have EHTs, while the health centres/ posts do not have an EHT due to staffing limitations. An in-charge at the health centre/post may be expected to take on more duties by acting as the environmental health focal point person but they may not have all the knowledge of HCWM that an EHT would. This may explain the lower rates of training observed for those at the health centres/posts compared to the district and regional hospitals. Special effort should be focused on the hospitals and health facilities without EHTs to ensure their staff are properly trained in HCWM. Training health workers is critical for effective waste management as this has a bearing on waste segregation, storage, collection, transportation and disposal [21]. Prior research has shown that training health workers improves knowledge, attitudes and practice for reducing hazards from infectious wastes [21,22].

With regard to preventing health hazards as a result of handling hazardous waste, many health facilities did not have full PPE for their waste management staff, with the lowest rate reported from the health centres/ posts (34%). Furthermore, needle sticks were alarmingly high as almost one-third (31%) of all health workers reported a needle stick injury while at work. A similar rate of needle stick injuries (43%) was found among health workers in Ethiopia [23]. A needle stick can expose the health care worker to various infectious diseases, such as HIV, Hepatitis B and C infections [10,11]. In Africa, medical waste handlers are more likely to contract Hepatitis B infection compared to medical waste handlers in non-African settings [24]. In Zambia, there is currently no policy to vaccinate all health workers, which raises the risk of Hepatitis B and other infections to the health care staff. There is a paucity of research on needle stick injuries in Africa [25], but our study shows a high rate of needle sticks among health workers in Zambia. In order to prevent needle stick injuries among health workers, sufficient PPE should be provided and training on the handling of sharp instruments should be given at orientation to new staff and at regular intervals thereafter [26].

It is particularly difficult to implement a safe and environmentally-friendly HCWM system in developing countries [2]. Inadequate funding, poor training and lack of awareness of policies and guidelines on HCWM have led to poor handling and disposal of medical waste in health facilities throughout Africa [2]. Zambian health facilities face these same challenges, especially the health centres/ posts. The Zambian Ministry of Health has developed HCWM guidelines and policies that are available at the provincial and district offices, but they were not always present at the health facilities, especially the health centres/ posts. In a study conducted in two South African hospitals, gaps were identified between the policy and implementation of HCWM practices, which ultimately led to poor waste management [27]. Having the Ministry of Health’s HCWM guidelines available at each health facility would allow health workers to review the guidelines and help them carry out proper health care waste handling, transport and disposal.

There were a few limitations in this study. One limitation is that we had to resort to a non-random sampling of health facilities during the data collection period due to logistical challenges accessing a few facilities. Also, some key-informants for the qualitative interviews were not available at the time of the visit for an interview, so we did not complete all targeted interviews. A fundamental limitation of this analysis is the inability to directly assess the individual practice of the health care workers. The practice analysis was based on self-report or the presence of certain HCWM items at the facilities rather than observing the actual practice of health workers, which may have biased our results. Lastly, there was a small representation of some health professionals surveyed, including doctors and midwives, which precludes generating conclusive findings regarding differences in HCWM training and knowledge between different health professions.

## Conclusions

Management of health care waste remains below national and international standards in various levels of health facilities in Zambia. The findings of this study indicate poor health care waste segregation, treatment and disposal, mainly due to a lack of proper HCWM tools, especially at the health centres/posts. Additionally, a high rate of needle sticks among health care workers was found. Based on the present study, we have identified three recommendations to improve HCWM in Zambia. The Ministry of Health should ensure that all health care workers undergo comprehensive training in the basics of health care waste management. Second, there is a need to include health care commodities (bins, bin liners, personal protective equipment, sharp containers/boxes) in the procurement process for medical and non-medical logistics. Lastly, we advocate for mainstreaming health care waste management and infection prevention, and control practices in all the different health programs.

## Supporting information

S1 Checklist Health care waste management assessment checklist. (DOCX)

S1 Text Questionnaire for knowledge, attitude, and practice survey for health care waste management study. (DOCX)

S2 Text Interview guide for KAP survey for health care waste management. (DOCX)

S3 Text Sample size calculation. (DOCX)
