# Supplementary material for: Knowledge, attitudes, and practices of health care waste management among Zambian health care workers
Source: PLOS Glob Public Health. 2022 Jun 22;2(6):e0000655. doi: 10.1371/journal.pgph.0000655 (PMC10021635; doi:10.1371/journal.pgph.0000655)
Supplement: S1 Checklist — (DOCX) [file pgph.0000655.s001.docx]

**S1 Checklist. Health Care Waste Management Assessment Checklist**

|  | **Name of the facility:** | | | **Available** | **Functional** |
| --- | --- | --- | --- | --- | --- |
|  | **Location:** | | |  |  |
|  | **Total number of workers:** | | |  |  |
|  | **Date of assessment:** | | |  |  |
| 1 | Is there a designated person in charge of Health Care Waste Management in the facility? | | |  |  |
| 2 | Is there written Standard Operating Procedures (SOPs) for waste management in the facility? | | |  |  |
| 3 | System of waste segregation of waste in place? | | |  |  |
| 4 | Are auto-disable syringes used for injection? | | |  |  |
| 5 | Are syringes discarded with their attached needles? | | |  |  |
| 6 | Do you have bins for disposing off infectious waste? | | |  |  |
| 7 | Do you have bins for disposing off non-infectious waste? | | |  |  |
| 8 | Syringes and needles disinfected before final disposal? | | |  |  |
| 9 | Is there an incinerator in a secured (fenced) site at this facility? | | |  |  |
| 10 | Do you have safety boxes at this facility | | |  |  |
| 11 | Do you have bin-liners at this facility | | |  |  |
| 12 | Categorise by colors | Yellow/Red | |  |  |
|  |  | Black | |  |  |
|  |  | Brown | |  |  |
|  |  | Others (indicate color) | |  |  |
| 13 | Is there a secured waste storage area on site? | | |  |  |
| 15 | Is there a specific budget for waste management? | | |  |  |
| 16 | Is there a functional IPC/HCWM committee? | | |  |  |
| **General Observations/Comments:** | | | | | |
| **Recommendations:** | | | | | |
| Names of Assessor(s): | | |  | | |
| Designation: | | |  | | |
| Date: | | |  | | |
| Signature: | | |  | | |
