# Supplementary material for: Knowledge, attitudes, and practices of health care waste management among Zambian health care workers
Source: PLOS Glob Public Health. 2022 Jun 22;2(6):e0000655. doi: 10.1371/journal.pgph.0000655 (PMC10021635; doi:10.1371/journal.pgph.0000655)
Supplement: S1 Text — (DOCX) [file pgph.0000655.s002.docx]

**S1 Text. Questionnaire for Knowledge, Attitude, and Practice survey for Health Care Waste Management Study.**

**Have you administered the informed consent?**

Yes/ No

**Please enter the questionnaire ID**

**Date/ Time**

**Name of Field Investigator: Select**

**Province: (select)**

**District: (select)**

**Health facility (select)**

Health Personnel-specific information:

What is the position of the person you are interviewing?

1. Doctor
2. Environmental health Staff
3. Community Health Assistant
4. Nurse
5. Clinical officer
6. Cleaner
7. Laboratory staff
8. Others (specify)……………………………….

Age:

Gender:

Years in service:

Type of hospital (tick one): (training/provincial) Regional District Sub-district

No. of inpatients: ____________/day No. of outpatients: ____________/day

No. of beds (total): ____________ Capacity **999= unknown**

1. Have you received any training on health care waste management?

Yes / No

2. Does your facility have waste management staff?

Yes / No

3. Does the waste management staff have job descriptions detailing their tasks?

Yes / No

4. Does the waste management staff have full personal Protective Equipment? (Gum Boots, coverall, heavy duty gloves)

Yes / No

5. Are instructions/training given to newly hired waste management staff? Yes No

**Hospital waste management policy**

1. Are you aware of any guidelines/policy for health care waste management? Yes No

3. Is there a manual or guideline document on management of hospital wastes available:

(a) In the Ministry of Health?

Yes No

(b) In your hospital?

Yes No

4. (a) Does your hospital have a Waste Management Plan?

Yes No If yes, please attach a copy.

5. Are there clearly defined procedures for collection and handling of wastes from specified units in the hospital?

Yes / No

6. Are there waste management responsibilities included in the job descriptions of hospital supervisory staff (Head of Hospital, Department Heads, Matron/Senior Nursing Officer, Hospital Engineer?

Yes / No

**Knowledge of waste segregation**

2. Does your health facility segregate waste?

Yes / No

**Health care waste Practices**

1. In what kind of storage facility do you place your waste in your working area?
2. Closed container with bin liner
3. Open container
4. No container, just in bag
5. Other:
6. How often does your storage facility get emptied for disposal?
7. Within 24 hours
8. More than 24 hours
9. Where do you place infectious waste?
10. In a yellow bag/bin
11. In a black bag/bin
12. It is mixed with other waste
13. Other (specify)……………………………..
14. What colour scheme does your health facility use to manage waste?
15. Black, Yellow, Red
16. Black, Yellow, Brown’
17. Red, Yellow, Black, Brown
18. Other
19. None
20. How is waste transported to the disposal site? (Multiple responses allowed)
21. In wheeled bins
22. Bins transported by hand by workers
23. Wheel barrows or Trolleys
24. Other

In what container should the following items be placed? *Do not read options

|  | Yellow/red Bag | Black bag | Sharps container |
| --- | --- | --- | --- |
| 1. Blood saturated gauge | X |  |  |
| 1. Empty IV bag |  | X |  |
| 1. Used hypodermic needle |  |  | X |
| 1. Suction canister with body fluids | X |  |  |
| 1. Broken mercury thermometer |  |  | X |
| 1. Used gloves | X |  |  |
| 1. Leftover food |  | X |  |

*Level of knowledge:*

*YES: High (6-7)*

*NO: Low (0-5)*

**Risks from health care waste management**

1. Have you had a needle prick before?

a) Yes

b) No

2. If yes, when did you have a needle stick injury?

1. Within 24 months on the job
2. Above 24 months on the job

3. Did you report your injury to management for mitigation?

1. Yes
2. No

Thank you for participating in this interview. Do you have any questions?

Note: for other comments/ questions.
