# Supplementary material for: Knowledge, attitudes, and practices of health care waste management among Zambian health care workers
Source: PLOS Glob Public Health. 2022 Jun 22;2(6):e0000655. doi: 10.1371/journal.pgph.0000655 (PMC10021635; doi:10.1371/journal.pgph.0000655)
Supplement: S2 Text — (DOCX) [file pgph.0000655.s003.docx]

**S2 Text. Interview Guide for KAP Survey for Health Care Waste Management**

**Remember to:**

1. Introduce Yourself.
2. Explain purpose of the survey.
3. Indicate that you would like to record and ask if they are okay with it.
4. Obtain consent.
5. Thank the respondent after the interview.
6. **BACKGROUND INFORMATION**
7. Name of Facility ………………………………………………
8. What is your formal training?
9. Briefly tell us about yourself in relation to the roles and responsibilities at this facility (ask main tasks,
10. What mechanism do you have in place to deal with healthcare waste management?
11. *Is there a committee/what are their roles?*
12. *What are some of the factors or issues around HWM that they prioritize?*
13. **POLICY AND PLANNING**
14. Does the healthcare facility have written policies, written plans, manuals, or written procedures dealing with healthcare waste management?
15. *What are the available polices?*
16. *Explain how they have been implemented and their effectiveness*
17. *Staff educated on them*
18. *What measures are in place to ensure their effectiveness and sustainability*
19. Are the policies, plans, manuals, and/or written procedures consistent with national laws, regulations, and any permits?
20. *How have they been applicable in your district/province*

1. Does the province/district/ facility have an annual allocation in its budget for healthcare waste management?
2. *Has it been sufficient?*
3. *Any projects implemented out of these funds to promote HCWM?*
4. **TRAINING AND CAPACITY BUILDING**
5. Have you ever received formal/technical training in HCW?
6. *If yes, provide details of what kind of training it was and how often you have received it.*
7. Does the province/district/ facilities have a training program on healthcare waste management for managers, health professionals, waste workers, and auxiliary staff? ***(Probe what trainings are in place)***
8. *who provides the training, duration and subjects covered?*
9. Are there areas of concern for training and capacity development that you think should be addressed?
10. What do you suggest should be done to improve the situation vis-à-vis training and capacity development?

**D. MONITORING AND EVALUATION and RECOMMENDATIONS**

1. Is there a system of internal monitoring or inspection to determine compliance with healthcare waste management requirements?
2. *What are their main roles and responsibilities?*
3. *The available policies/plans are they reviewed and how often*
4. *the measures/plans/policies implemented that they can point out to be effective (probe on how)*
5. Is there a system of taking corrective action when practices or technologies related to healthcare waste management do not meet the requirements? (*probe how it operates*)
6. What measures/recommendations do you think need to be put in place towards KAP on health care waste management?

***END OF THE INTERVIEW***

***THANK YOU VERY MUCH FOR YOUR VALUABLE TIME!***
