# Supplementary material for: Knowledge, attitudes, and practices of health care waste management among Zambian health care workers
Source: PLOS Glob Public Health. 2022 Jun 22;2(6):e0000655. doi: 10.1371/journal.pgph.0000655 (PMC10021635; doi:10.1371/journal.pgph.0000655)
Supplement: S3 Text — (DOCX) [file pgph.0000655.s004.docx]

**S3 Text. Sample size calculation**

# Quantitative Sampling:

The sample size will be calculated using the formula below:

Sample size calculation


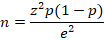

 is the statistic that defines the level of confidence, in this case the z value will be 1.96.

 Is the proportion used in the estimation formula, in this study p is 50% because there is currently no data on prevalence of the knowledge, attitudes and practices of health care workers in health care waste management.

 Is a measure of precision, thus the margin of error. In this study the margin of error is set at 0.05

Here is the estimated sample size:


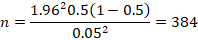


Adjusting the sample size upwards for assumed non-response rate () then the sample size will be adjusted as follows:

Where is the final sample size and is the expected response rate in decimals which is 93.75% (0.9375) in accordance with the ZDHS of 2013-2014, (Zambia Central Statistics Office, 2015).

410

9375

.

0

384

≈

=

*f*

*n*
